# Bioinformatics Profiling of Five Immune-Related lncRNAs for a Prognostic Model of Hepatocellular Carcinoma

**DOI:** 10.3389/fonc.2021.667904

**Published:** 2021-05-28

**Authors:** Fahong Wu, Hangzhi Wei, Guiyuan Liu, Youcheng Zhang

**Affiliations:** Department of General Surgery, Hepatic-biliary-pancreatic Institute, Lanzhou University Second Hospital, Lanzhou, China

**Keywords:** hepatocellular carcinoma, prognosis, T cell, immune lncRNAs, cell cycle

## Abstract

Hepatocellular carcinoma (HCC), one of the most common tumors worldwide, has the fifth highest mortality rate, which is increasing every year. At present, many studies have revealed that immunotherapy has an important effect on many malignant tumors. The main purpose of our research was to verify and establish a new immune-related lncRNA model and to explore the potential immune mechanisms. We analysed the pathways and mechanisms of immune-related lncRNAs by bioinformatics analysis, screened key lncRNAs based on Cox regression analysis, and determined the characteristics of the immune-related lncRNAs. On this basis, a predictive model was established. Through a comparison of specificity and sensitivity, we found that the constructed model was superior to the known markers of HCC. Then, the cell types were identified by the relative subgroup (CIBERSORT) algorithm for RNA transcripts. A signature model was eventually constructed, and we proved that it was a survival factor for HCC. Moreover, five kinds of immune cells were significantly positively correlated with the signature. The results indicated that these five kinds of lncRNAs may be related to the immune infiltration of hepatocellular carcinoma. To verify these findings, we selected the top coexpressed lncRNA, AC099850.3, for further study. We found that AC099850.3 could promote the migration and proliferation of hepatocellular carcinoma cells *in vitro*. RT-PCR experiments found that AC099850.3 could promote the expression of the cell cycle molecules BUB1, CDK1, PLK1, and TTK, and western blotting to prove that the expression of the molecules CD155 and PD-L1 was inhibited in the interference group. In conclusion, we used five kinds of immune-related lncRNAs to construct prognostic signatures to explore the mechanism, which provides a new way to study therapies for HCC.

## Introduction

Hepatocellular carcinoma (HCC) has the fifth highest fatality rate in the United States and is one of the five deadliest cancers, with the percentage increasing each year (1). In our country, the fatality rate of HCC is higher than that of developed countries (2). The prognosis of HCC is worse. Few patients can undergo surgical resection, and there is usually no liver cirrhosis due to impaired liver regeneration (3). Given the limitations and adverse reactions of HCC treatment, new treatments are urgently needed. At present, many studies have revealed that immunotherapy has an important effect on many malignant tumors (4–6). Immunotherapy usually targets stem cells to prevent resistance to chemotherapy (7). Another method is to use PD-1 and PD-L1 to suppress the immune system and tumor cell escape, thus affecting the occurrence and development of tumors. Sorafenib can antagonize the effect of immunosuppressors, so it may be effective in combination with tumor therapy (8, 9). Therefore, we need to study the immune infiltration mechanism of HCC, which is important for developing a new approach for tumor immunotherapy.

Given the poor prognosis of patients with HCC, we urgently need to find new prognostic immune-related markers of HCC. Bioinformatics analysis enables researchers to analyse multiple databases and large samples, deepening the communication among researchers (10). In this context, researchers can more deeply study the comprehensive data of many clinical samples from different independent studies, which provides a basis for the discovery of promising immune biomarkers of HCC through the use of bioinformatics analysis. We are more likely to find new immune markers of HCC in a cost-effective manner and identify the shortcomings of known biomarkers of liver cancer (11–13).

In our study, we established a risk model based on immune-related lncRNAs, which could independently predict the survival of patients with HCC. Then, the model was combined with clinical characteristics to assess the relationship between them, and the relationship between the model and infiltrating cells was examined. In addition, the regulatory pathways of immune-related lncRNAs in HCC were revealed by GSEA. Finally, to verify the function, we selected the top coexpressed lncRNA, AC099850.3, for further study. The results showed that AC099850.3 could promote the migration and proliferation of hepatocellular carcinoma cells *in vitro*, and RT-PCR experiments found that AC099850.3 could promote the expression of the cell cycle molecules BUB1, CDK1, PLK1, and TTK. Western blotting to prove that the expression of the molecules CD155 and PD-L1 was inhibited in the interference group.

## Methods

### Data Source

RNA-sequencing data were obtained from the UCSC Xena platform, The Cancer Genome Atlas (TCGA) and the Genotype-Tissue Expression (GTEx) project, and the raw count data of 374 HCC samples and 160 nontumor samples were collected, and the Limma R package was used to reduce the batch effect for the merger. In addition, we obtained all the clinical features of the patients from TCGA database and downloaded immune genes from the molecular signature database (MsigDB) (https://www.gsea-msigdb.org/gsea/msigdb/index.jsp). The “DESeq2” software package in R software was used to normalize the RNA expression profile, and stable transformation of the variance was carried out. This study was conducted in accordance with TCGA’s publishing guidelines and data access policies (http://cancergenome.nih.gov/publications/publicationguidelines).

### Screening of Immune-Related lncRNAs

We used the DESeq2 software package in R software to detect differentially expressed mRNAs (DEMs) in HCC and normal tissues, and we obtained 2867 DEMs according to the standard (P < 0.01 and |log2FC| > 1). Then, we identified 59 immune-related mRNAs from the DEMs by MsigDB (14), and 401 immune-related lncRNAs were obtained by coexpression analysis (P < 0.01 and |Cor| > 0.3). The “ggplot2” software package of R software was used to generate the volcano map, and the coexpression network map was made with Cytoscape software.

### Proven Prognostic Relevance of lncRNAs in HCC

We used univariate CPHR analysis and the Kaplan-Meier method. When the results from the two methods were consistent, the lncRNAs were identified as OS-related lncRNAs. Removing samples with no clear clinical features, we obtained 337 HCC patients (“the entire dataset”). Of the 337 patients with HCC, 169 were randomly designated as the “training dataset”, and 168 patients were defined as the “test dataset”. Importantly, there were no statistically significant clinical differences between the two datasets. Across the entire dataset, candidates for OS-related lncRNAs were selected for multivariate studies conducted with SPSS software (stepwise model). We calculated the AIC and used it to evaluate a statistical model for a given data set and took the minimum AIC value as the optimal prediction model.

### Construction of a Risk Score Model for Five Immune-Related lncRNAs

To obtain the best-fit OS-related lncRNAs of HCC, we carried out univariate Cox regression analysis to determine the relationship between the expression level of lncRNAs and the OS of patients, and lncRNAs were considered to be statistically significant with a cut-off value of P < 0.001. We selected OS-related lncRNAs with statistical significance in the univariate Cox regression analysis and then performed Lasso Cox regression analysis on this basis. If the differentially abundant lncRNAs were statistically significant in the univariate analysis and multivariate analysis, we could consider these lncRNAs independent prognostic factors of HCC (15). LASSO and multivariate Cox regression analysis was conducted to calculate the coefficients of each OS-related lncRNA. Therefore, we constructed a risk score formula:

$$\begin{array}{c} riskScore=Expression_{mRNA1}\times Coefficient_{mRNA1}+Expression_{mRNA2}\\ \times \;Coefficient_{mRNA2}+\ldots Expression_{mRNAn}\times Coefficient_{mRNAn.} \end{array}$$

To prove the predictive ability of the model, we used the “survival” package in R to perform Kaplan-Meier (KM) survival analysis to reveal the OS of high-risk and low-risk groups. Time-dependent ROC curves were constructed, and AUC values for 1 and 3 years were calculated. In addition, we conducted a hierarchical analysis to evaluate whether the relationship between the risk score models and OS was independent of clinical risk factors (16).

### A Relationship Between the Model and Immune Cells

To predict the immune cells related to the model, we used the genes to calculate the proportion of 22 immune cells by the CIBERSORT web portal (https://cibersort.stanford.edu/index.php) (17). We obtained obvious immune cell types and the distinction of immune cells between tumor tissues and normal tissues and researched the relationship between immune cells and five immune-related lncRNAs to assess the prognosis of patients. Finally, using Pearson correlation analysis, we generated a heat map of coexpression to show the relationship between 22 immune cells and the five immune-related lncRNAs.

### GO and KEGG Pathway Enrichment Analyses

Pearson correlation tests were used to assess the coexpression of the five lncRNAs and mRNAs (|cor| > 0.3). Only mRNAs with differential expression in coexpression results were selected for further enrichment analysis to reduce false positives. To reveal the function of the five lncRNAs, R software was used for enrichment analysis of Gene Ontology (GO) terms and KEGG pathways 10. Adj. P<0.05 was considered statistically significant. In addition, we divided the constructed model into high-risk and low-risk groups and carried out gene set enrichment analysis (GSEA) to reveal signal transduction (http://software.broadinstitute.org/gsea/).

### Cell Lines and siRNA Transfection

Human HCC cell lines (HCCLM9, Huh7 and HepG2) and the normal liver cell line LO2 were obtained from the Institute of School of Pharmacy, Lanzhou University and cultured separately in DMEM with 10% FBS (Gibco, Grand Island, NY, USA). AC099850.3 shRNA and negative control (NC) shRNA oligonucleotides were designed by FENGHUISHENG WU (Changsha, China). The lncRNA AC099850.3 sh1 and sh2 sequences were 5’-GGAGCTGACATTTAAACCAAGG-3’ and 5’-GCAACAGTATACAAGACATCC-3, respectively. According to the instructions, when the cells reached 30%-50% confluence, Lipofectamine 2000 (Life Technologies) was used to transfect the interfering group in OptiMEM medium.

### Quantitative Real-Time Polymerase Chain Reaction and Western Blotting

Cells transfected with vectors were lysed with lysis buffer at 4°C for 30 min. The concentration of cells was measured by the Nanodrop2000 (Thermo Fisher Scientific). Total RNA was extracted from cells with TransZol UP reagent (Transgene, Strasbourg, France) and reverse transcribed with the Revert Aid First Strand cDNA Synthesis Kit (Servicebio). Primer sequences are detailed in **Supplementary Table 1**. PCR was performed with Fast Start Universal SYBR Green Master Mix (Roche, Basel, Switzerland), and the fluorescence was measured using an CFX96 Real Time System (Applied Biosystems, Life Technologies, Foster City, CA, USA) by following the manufacturer’s instructions. Data were analyzed using the 2−ΔΔCt method, and β-actin was regarded as an internal control.

Cells transfected with vectors were lysed with lysis buffer at 4°C for 30 min. The concentration of cells was measured by the BCA Protein Assay Kit (Beyotime Institute of Biotechnology, Shanghai, China). Proteins were separated by SDS-PAGE and transferred to polyvinylidene fluoride membranes, which were then sealed with non-fat milk in tris-buffered saline with Tween for 2 h. The appropriate diluted primary antibodies, including anti-human Tubulin (1:2000; Invitrogen Carlsbad, CA, USA), anti-human CD155 (1:1000; Cell Signaling Technology, Danvers, MA, USA), anti-human PD-L1 (1:1000; Cell Signaling Technology), were then incubated with the membranes overnight at 4°C. The second antibody was diluted with TBST and incubated with the membranes for 1 h at room temperature. Immunoreactivity was determined using a chemiluminescence western blot immunodetection kit (Invitrogen) according to the manufacturer’s instructions. The amounts of each protein were semi-quantified, and Tubulin was regarded as an internal control.

### Transwell Assay

Cell migration was analysed by 24-well Transwell chambers with 8 μm polycarbonate membranes (Millipore, Washington, DC, USA). The cells were inoculated with serum-free DMEM at 2×10^5^ cells per well in the upper cavity, and 12% FBS was added to the lower cavity. After incubation for 48 hours, the cells were fixed with paraformaldehyde and stained with crystal violet. The cells in the lower chamber were counted according to 5 random regions. Each assay was performed in triplicate.

### Cell Proliferation Assay

According to the manufacturer’s instructions (Servicebio), we used Cell Counting Kit-8 (CCK-8) assays to determine the proliferation of HCC cells. In short, the 96-well plate was inoculated with 2×10^3^ cells/well, 10 µl of CCK-8 and 90 µl of culture medium reagent were added to each well, and after 1.5 h incubation at 37°C, the OD values at 24 h, 48 h, 72 h and 96 h were detected at wavelengths of 450 nm and 600 nm. Colony formation experiments were performed. A total of 1×10^3^ cells/well were inoculated in 6-well plates for 7 days. The plate was fixed with methanol and then was stained with crystal violet solution after 30 min.

### Data Analysis

We used R version 3.5.1 software to analyse our data (Institute for Statistics and Mathematics, Vienna, Austria; www.r-project.org) (18). A P value< 0.05 was considered statistically significant.

## Results

### Data Source and Screening of Immune-Related Candidate lncRNAs

**Figure 1:**
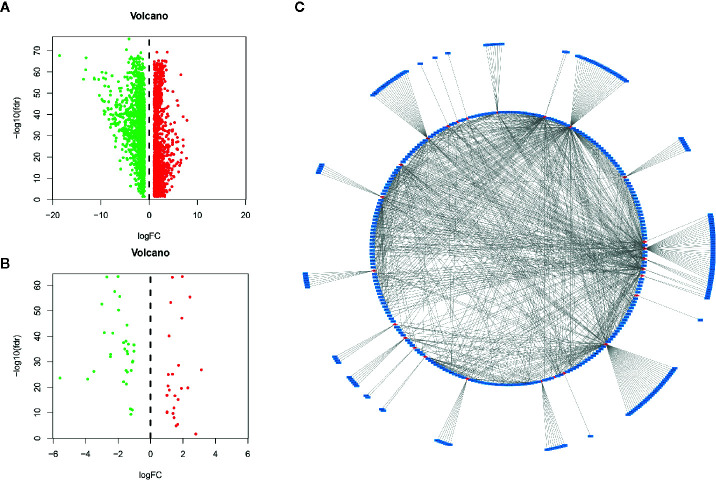
Identification of lncRNAs related to immune genes by coexpression analysis. **(A)** Differentially expressed genes were expressed in hepatocellular carcinoma and normal tissues, and **(B)** 59 immune-related genes were obtained by further analysis. Green and red points indicate downregulated and upregulated lncRNAs in volcano plot, respectively. **(C)** Coexpression analysis of lncRNAs associated with immune-related mRNAs, and obtained 401 immune-related lncRNAs (P < 0.01 and |cor| > 0.3, red for mRNAs, blue for lncRNAs).

A total of 534 samples were collected from TCGA and GTEX databases (160 normal samples and 374 HCC samples). As shown in **Figure 1A**, we obtained 2867 DEmRNAs according to the standard (P < 0.01 and |log2FC| > 1). Moreover, we analysed and obtained 59 immune genes from the DEmRNAs (**Figure 1B**), and then, we used coexpression analysis to obtain 401 immune-related lncRNAs (**Figure 1C**, P < 0.01 and |cor| > 0.3). New prognostic lncRNAs related to HCC were screened by univariate analysis (**Supplementary Table 2**), and then, we removed patients without clinical data and obtained 337 HCC samples (entire dataset). Of the 337 patients with HCC, 169 were randomly designated as the “training dataset”, and 168 patients were defined as the “test dataset” (**Table 1**). The (13) lncRNAs were evaluated by the LASSO regression (**Supplementary Figure 1**) and Cox proportional hazard model in the R package survival analysis to find the best model in the training dataset. In addition, multivariate analysis identified five immune-related lncRNA risk scores for HCC as follows: (**Supplementary Table 3**)

$$\begin{array}{c} Riskscore=AC009005.1*0.517+AC129492.1*-0.963+AC099850.3*0.473\\ +AL365203.2*0.739+AC015908.3*-1.844 \end{array}$$

**Table 1:** Clinical characteristics of 337 patients with hepatocellular carcinoma.

Character	Test dataset Training dataset		Entire dataset	P value
	n=168	n=169	n=337	
**Age**				0.534
≥65	60	70	130	
<65	109	98	207	
**Gender**				0.610
Female	56	51	107	
Male	107	123	230	
**TNM stage**				0.244
I-II	112	138	250	
III-IV	57	30	87	
**Tumor stage**				0.961
T1-T2	128	125	253	
T3-T4	41	43	84	
**Distant metastasis**				0.898
Mx	36	40	76	
no	130	228	258	
yes	2	1	3	
**Grade**				
G1-G2	102	109	211	0.692
G3-G4	67	59	126	

### Development and Analysis of Five Immune-Related lncRNA-Based HCC Model Assays

There was no significant difference in the clinical characteristics between the three groups of patients (all P > 0.05), indicating that the training dataset can represent the test dataset.

**Figure 2:**
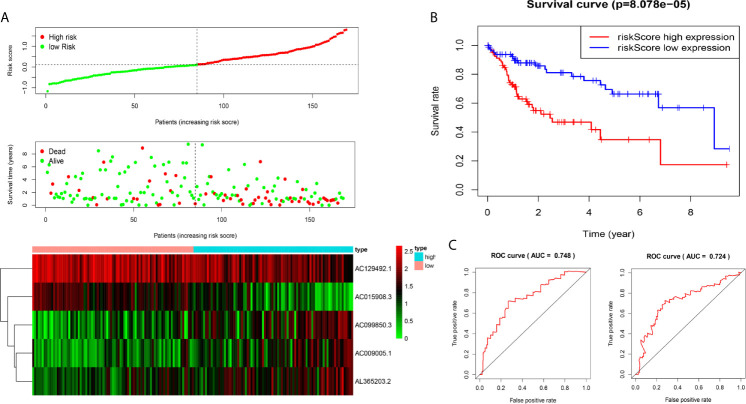
Construction of five-immune-related gene models from the training dataset. **(A)** Distribution of high-risk and low-risk groups of patients, scattergram of the patients in the two groups, and heat map of the expression of five lncRNAs in HCC. **(B)** Kaplan-Meier curves for the OS of patients in the high-risk group and low-risk group in the training dataset. **(C)** The area under the time-dependent ROC curve (AUC) of time-dependent receiver operating characteristic (ROC) curves verified the prognostic performance of the risk score in the training dataset, and the results showed that the AUC of the model was 0.748 at one year, 0.724 at three years.

We analysed the risk score of each HCC patient in the traning dataset (n=169). Based on outcomes in line with the median, HCC samples were divided into two groups. The outcomes revealed the risk score, OS status and distribution in the dataset (**Figure 2A**). The heat map showed that the expression levels of the lncRNAs AC009005.1, AC099850.3 and AL365203.2 in the high-risk group were higher than those in the low-risk group, while the expression levels of AC129492.1 and AC015908.3 presented the opposite results. K-M analysis revealed that the survival rate of the high-risk group was poorer than that of the low-risk group (**Figure 2B**). Moreover, we established an ROC curve in the dataset, and the results showed that the area under the time-dependent ROC curve (AUC) of the model was 0.748 at one year, 0.724 at three years (**Figure 2C**).

**Figure 3:**
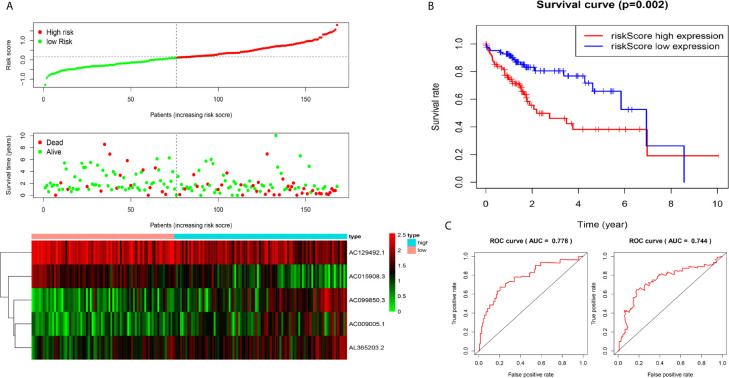
Construction of five-immune-related-gene models from the test dataset. **(A)** Distribution of high-risk and low-risk groups of patients, scattergram of the patients in the two groups, and heat map of the expression of five lncRNAs in HCC. **(B)** Kaplan-Meier curves for the OS of patients in the high-risk group and low-risk group in the test dataset. **(C)** AUC of time-dependent receiver operating characteristic (ROC) curves verified the prognostic performance of the risk score in the test dataset, and the results showed that the AUC of the model was 0.778 at one year, 0.744 at three years.

We used the test dataset (n=168) to further verify the ability of the 5-lncRNA models to predict survival and applied the five lncRNAs models and cut-off values of the training dataset to the test dataset. the distributions of the five lncRNAs based risk scores, OS statuses and five lncRNAs expression profiles in the test dataset were consistent with those described above (**Figure 3A**). Similar to the results in the test dataset, K-M analysis revealed that the survival rate of the high-risk group was poorer than that of the low-risk group (P=0.002) (**Figure 3B**), and the AUC of the model was 0.778 at one year, 0.744 at three years (**Figure 3C**).

### A Nomogram Combining Risk Score and Clinical Features Evaluated Survival in HCC

**Figure 4:**
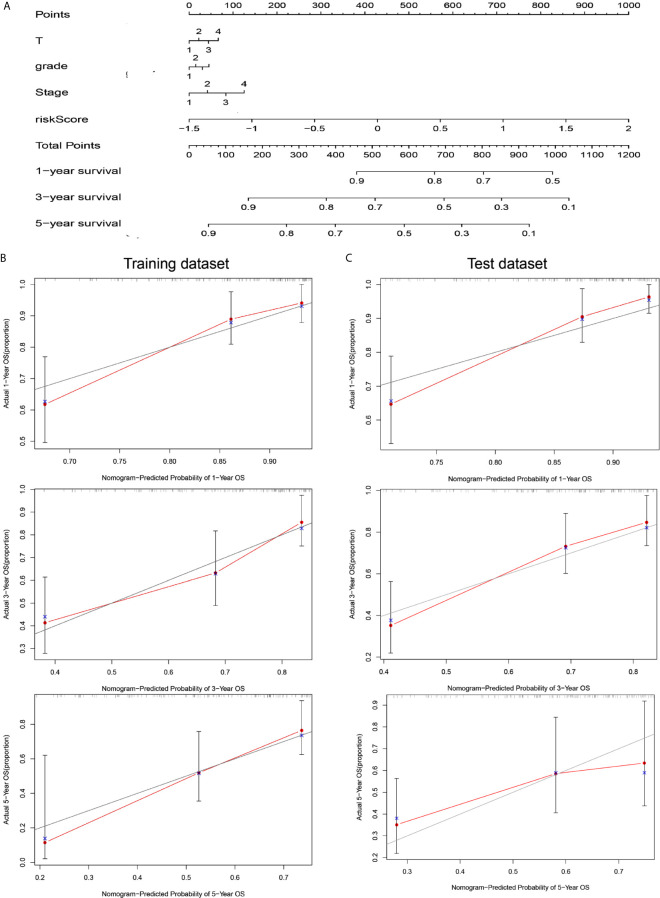
A nomogram combining risk score and clinical features evaluated survival in HCC. **(A)** Nomogram for predicting OS. Description: position each feature point on the corresponding variable axis, draw a vertical line upward to the point axis, and determine the value of the specific point. Repeat the process. Calculate the total score and position it on the total axis. Draw a vertical line down to the one-, three- and five- year OS to obtain the survival probability of specific liver cancer patients. **(B)** The calibration curve of the nomogram for one, three, and five years of OS prediction in the training dataset. **(C)** The calibration curve of the nomogram for one, three, and five years of OS prediction in the test dataset.

To coordinate clinical importance and statistical significance, we constructed nomograms based on a 5-immune-related-lncRNA signature, which integrated a five-lncRNA model and three clinical features (T, grade, and stage). Finally, we estimated the one-, three- and five-year prognoses of HCC patients in a nomogram (**Figure 4A**). The results showed that the risk score was closely related to OS, followed by T, grade and stage. Then, we used the concordance index and calibration plots to predict the differential and correction ability of the prognostic nomogram from the training dataset (**Figure 4B**). In the test dataset, we obtained similar results as those described above. The figure shows that the nomogram of the training dataset and test dataset were close to the actual probabilities (**Figure 4C**).

### Relationship Between the Five-lncRNA Signature and Clinical Characteristics

**Figure 5:**
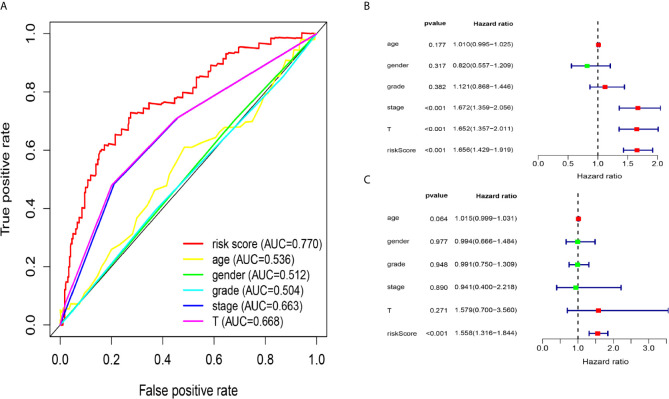
Relationship between the model and clinical characteristics in entire dataset. **(A)** The AUC values of clinical features and 5-immune-related lncRNAs were analysed, and the figure shown that the AUCs all of clinical characteristics were lower than those of the 5-immune-related lncRNA signatures **(B)** Univariate analysis suggested that the risk scores from the signature, T, and stage were associated with OS (stage: TNM stage, T: tumor stage) (P < 0.001). **(C)** Multivariate Cox analysis showed that our model could independently predict OS of the HCC patients (P < 0.001).

Due to the problem of sample size, we chose the entire dataset (The test and training data sets were included in **Supplementary Material Figure 2**) to explore the relationship between the five-lncRNA models and the clinical characteristics. Our studies used ROC curves to compare the sensitivity and specificity of different survival factors. As shown in **Figure 5A**, the AUCs of age, sex, grade, stage, and T stage were 0.536, 0.512, 0.504, 0.663, and 0.668, respectively, all of which were lower than those of the 5-immune-related lncRNA signatures (AUC=0.770). To verify whether the model could be employed as a survival indicator of OS, we performed univariate and multivariate Cox regression analyses on the model and clinical variables (including age, sex, grade, stage, and T stage). Our univariate analysis suggested that the risk scores from the signature (HR 1.656, 95% CI 1.429−1.919, P < 0.001), stage (HR 1.672, 95% CI 1.359−2.056, P < 0.001), and T stage (HR 1.652, 95% CI 1.357−2.011, P < 0.001) were associated with OS (**Figure 5B**). Some features with important distinctiveness in univariate analysis were used in multivariate analysis, which showed that our model (HR 1.558, 95% CI 1.316−1.844, P <0.001) could independently predict OS of the HCC patients (**Figure 5C**).

### The Relationship Between Immune Cells and Clinical Characteristics of HCC

**Figure 6:**
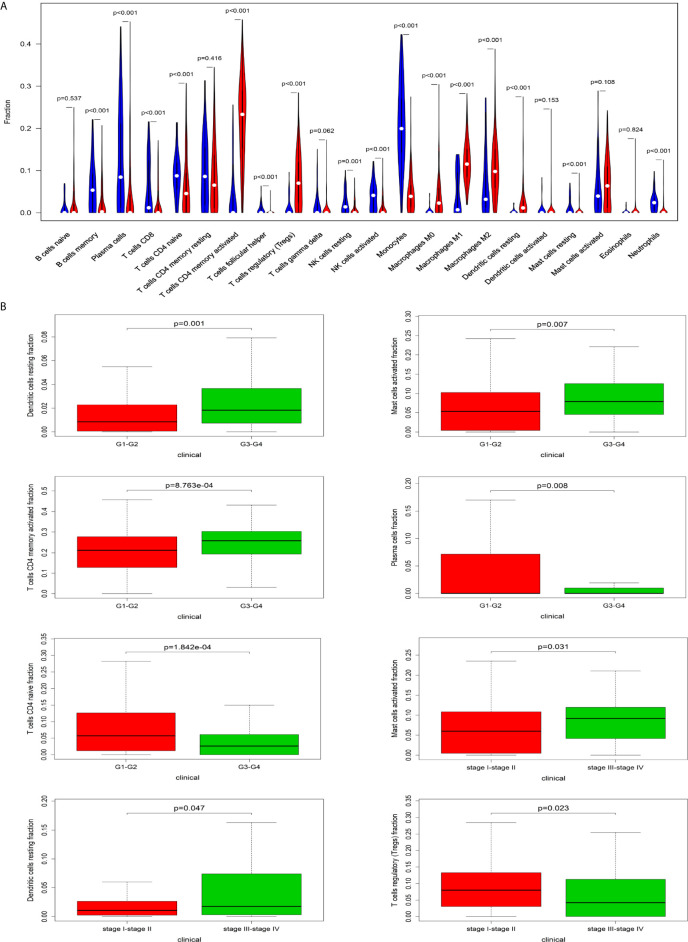
The relationship between immune cells and clinical characteristics in HCC. **(A)** The differences in the composition of tumor﻿-infiltrating immune cells between normal tissue and HCC tissues. CIBERSORT was employed to evaluate the compositions of immune cells in HCC and to identify immune cells related to HCC. **(B)** The box plots about grade and TNM stage of resting dendritic cells, activated mast cells, activated CD4 memory T cells, plasma cells, CD4 naive T cells, and regulatory T cells (P < 0.05).

The CIBERSORT algorithm was employed to evaluate the important tumor infiltrating immune cell components of HCC. As shown in **Figure 6A**, comparing HCC tissues with normal tissues, we found that there were significant differences in 16 immune cells, including memory B cells, plasma cells, CD8 T cells, naive CD4 T cells, active CD4 memory T cells, follicular helper T cells, regulatory T cells, resting NK cells, active NK cells, monocytes, M0 macrophages, M1 macrophages, M2 macrophages, resting mast cells and neutrophils (**Figure 6A**). Resting dendritic cells (G1-G2 *vs* G3-G4: P=0.001; stage I-II *vs* stage III-VI: P = 0.047), activated mast cells (G1-G2 *vs* G3-G4: P=0.007; stage I-II *vs* stage III-VI: P = 0.031), activated CD4 memory T cells (G1-G2 *vs* G3-G4: P<0.001), plasma cells (G1-G2 *vs* G3-G4: P=0.008), CD4 naive T cells (G1-G2 *vs* G3-G4: P<0.001), and regulatory T cells (stage I-II *vs* stage III-VI: P=0.023) all had obvious relationships with the clinical characteristics (**Figure 6B**).

### Relationship Between Immune Cell Infiltration and Signature Based on 5 Immune-Related lncRNAs

**Figure 7:**
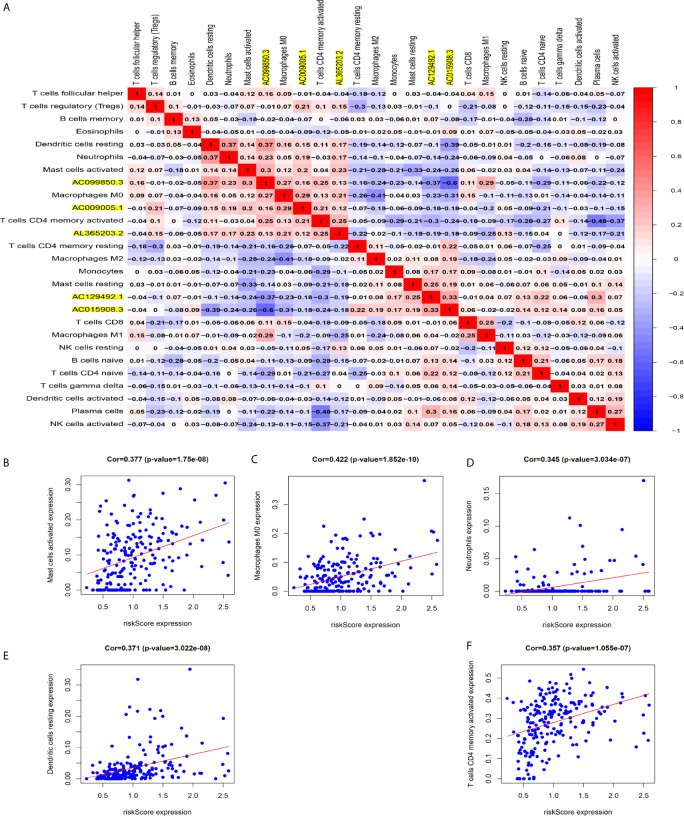
Relationship between immune cell infiltration and the model based on five immune-related lncRNAs. **(A)** The co-expression heat map shows some important co-expression patterns of members of five immune-related lncRNAs and key members of immune cells. **(B–F)** The relation between risk scores and infiltration of the five types of immune cells (activated mast cells, M0 macrophages, neutrophils, resting dendritic cells, and activated CD4 memory T cells).

**Figure 8:**
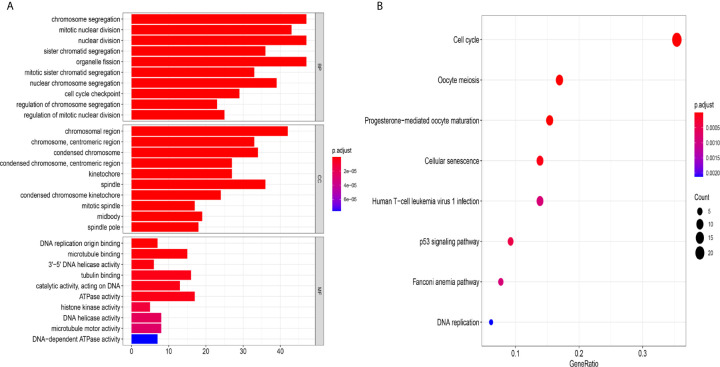
The mechanism of the five-lncRNA model. **(A)** GO enrichment analysis. MF, CC and BP words represented the GO terms for molecular functions, cellular components and biological processes, respectively. The y-axis represented the obvious enrichment path of model. The x-axis represented the gene numbers of model, and the color represented different p values. **(B)** The first 8 functional pathways related to these genes were revealed by KEGG analysis. The y-axis represented the obvious enrichment path of model. The enrichment factor on the x-axis indicated the degree of enrichment. The higher the value of the enrichment factor, the higher the degree of richness. The color of the dot represented different p values, and the size of the dot reflected the number of target genes enriched in the corresponding pathway.

**Figure 8A** shows some important coexpression patterns of important members of the five lncRNAs (**Figure 7A**). We found that five kinds of immune cells were significantly positively related to the signature: activated mast cells (Cor=0.337, P<0.001), M0 macrophages (Cor=0.422, P<0.001), neutrophils (Cor=0.345, P<0.001), resting dendritic cells (Cor=0.371, P<0.001), and activated CD4 memory T cells (Cor= 0.357, P<0.001) (**Figures 7B–F**).

### The Mechanism of the Five-lncRNA Model

To further explore the related mechanism of the five-lncRNA models in HCC, we analysed the functional enrichment of coexpressed mRNAs from 374 HCC samples in GO and KEGG pathways. A total of 295 coexpressed genes were screened (Pearson correlation coefficient > 0.40). GO (Gene Ontology) analysis showed that these genes were enriched in “cell cycle checkpoint”, “chromosomal region”, and “DNA replication origin binding” (**Figure 8A**), and these genes were also proven to be actively involved in the cell cycle according to KEGG enrichment analysis (**Figure 8B**). More importantly, we found these genes were enriched in human T-cell leukemia (1) infection in **Figure 8B**. These results suggested that the five-lncRNA model was associated with the development of HCC through the cell cycle and the immune mechanism.

### LncRNA AC099850.3 Promoted the Proliferation and Migration of HCC Cells

**Figure 9:**
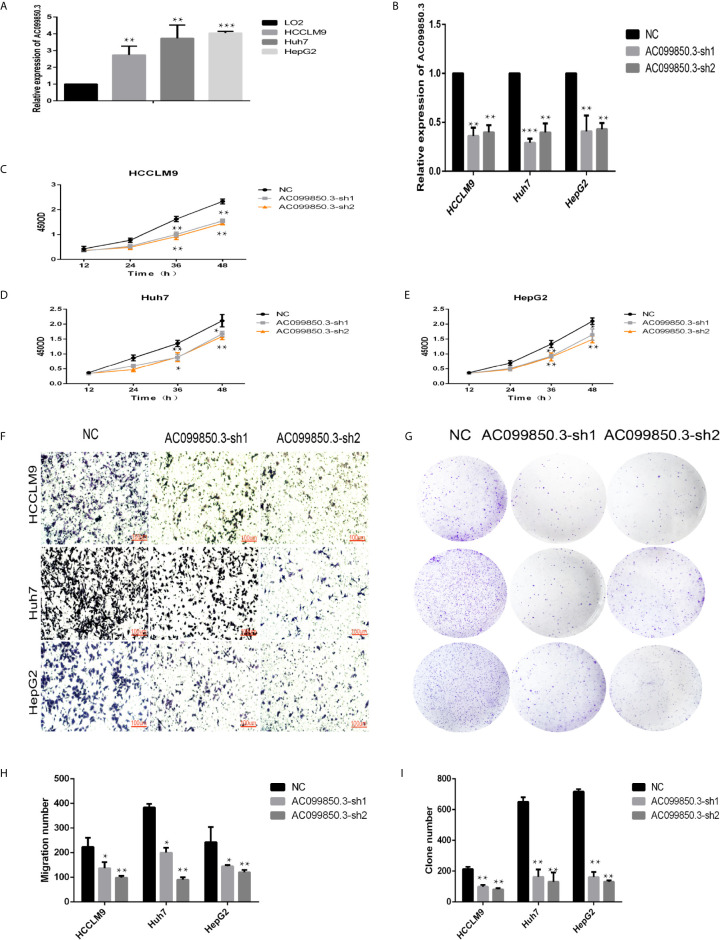
LncRNA AC099850.3 promoted the proliferation and migration of HCC cells. **(A)** The expression of AC099850.3 in HCCLM9, Huh7, and HepG2 cells was significantly higher than that in normal LO2 cells. **(B)** Verification of the AC099850.3 interference efficiency of the HCC cell line at the mRNA level. **(C–E)** The CCK-8 assay showed that the HCC cell activity in the interference groups was lower than that in the control groups. **(F)** Transwell analysis showed that cell migration was significantly inhibited in the interference groups. **(G)** Colony formation analysis showed that the proliferation rate of cells in the interference groups was significantly inhibited. **(H, I)** The histograms of the Transwell and the Colony formation analysis, respectively. (*P < 0.05, **P < 0.01, ***P < 0.001).

We chose AC099850.3, which had the most coexpression results (**Supplementary Table 4**), as the research object and discussed its related mechanism10. We demonstrated that AC099850.3 was highly expressed in HCC cells, and the knockout efficiency was verified at the mRNA level (**Figures 9A, B**). The viability of HCCLM9, Huh7, and HepG2 cells in the interference groups was significantly lower than that in the negative control group (**Figures 9C–E**). Transwell analysis showed that cell migration was significantly inhibited after target gene knockout (**Figure 9F**). Colony formation analysis showed that the proliferation rate of the cells in the interference group was significantly inhibited (**Figures 9G–I**) were histograms of the chamber experiment and the monoclonal experiment, respectively (**Figures 9H, I**). These results suggested that AC099850.3 promotes the proliferation and migration of HCC cells.

**Figure 10:**
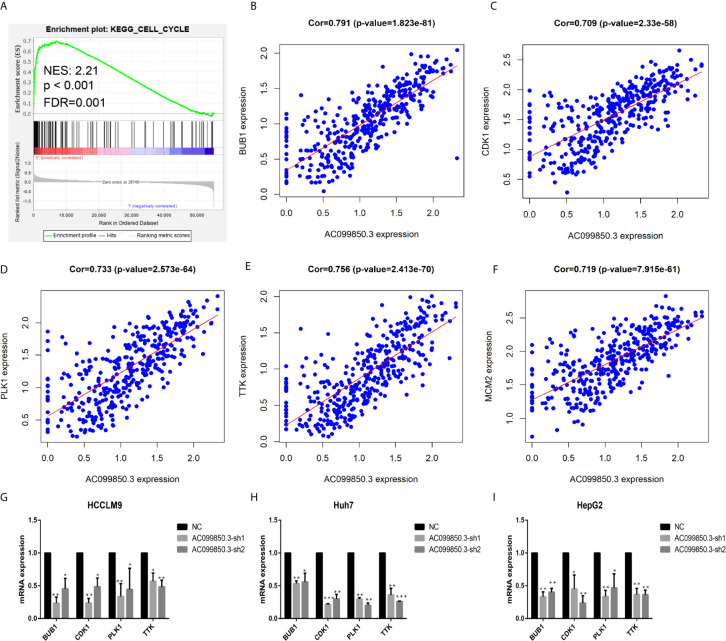
The relevant mechanism of AC099850.3. **(A)** GSEA showed that AC099850.3 was related to the cell cycle signalling pathway. The horizontal axis was each gene under the gene, and the vertical axis was the corresponding Running ES (enrichment score). There was a peak in the line chart, and the peak was the ES of the gene set. NES represented the normalized Enrichment score, and FDR q-val represented the p value corrected by multiple hypothesis tests. **(B–F)** Coexpression analysis predicted the AC099850.3-related cell cycle molecules BUB1, CDK1, PLK1, TTK, and MCM2 (cor > 0.7). **(G–I)** The results of the PCR assay showed that the interference groups had inhibited expression of the cell cycle molecules BUB1, CDK1, PLK1, and TTK. (*P < 0.05, **P < 0.01, ***P < 0.001).

**Figure 11:**
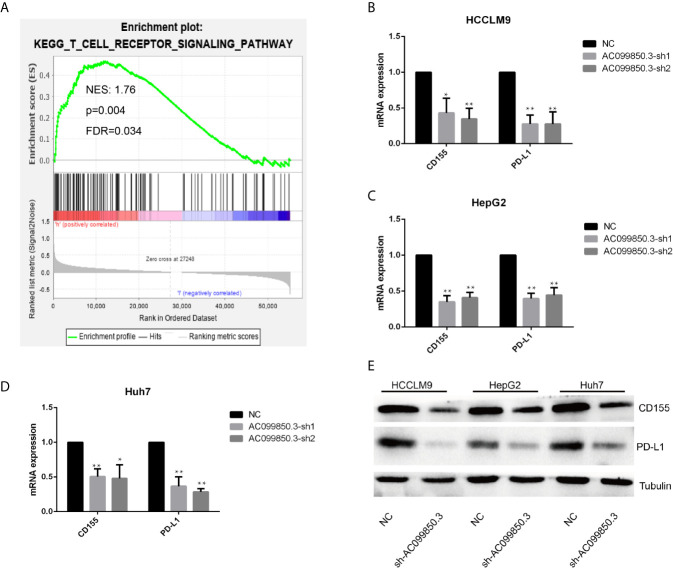
The relevant immune mechanism of AC099850.3. **(A)** GSEA showed that AC099850.3 was related to the T cell receptor signalling pathway. About the legend as described in the above Figure 10A. **(B–D)** The results of the PCR assay showed that the interference groups had inhibited expression of the molecules CD155 and PD-L1. **(E)** The results of the western blotting assay showed that the interference groups had inhibited expression of the molecules CD155 and PD-L1 in the HCC cell lines. (*P < 0.05, **P < 0.01).

To further study the relevant mechanism of AC099850.3, we used GSEA to analyse the signalling pathway, and we found that it was related to the cell cycle, which was consistent with the above functional analysis results (**Figures 10A**). Then, we used bioinformatics analysis to predict the main molecules of the cell cycle and found that AC099850.3 could regulate the expression of BUB1, CDK1, PLK1, TTK, and MCM2 (**Figures 10B–F**). Finally, we used PCR to verify that the expression of the cell cycle molecules BUB1, CDK1, PLK1, and TTK was inhibited in the interference group (**Figures 10G–I**). Then, to further study the immune relevant mechanism of AC099850.3, we used GSEA to analyse the signalling pathway, and we found that it was related to the T cell receptor (**Figure 11A**). We used PCR to verify that the expression of the molecules CD155 and PD-L1 was inhibited in the interference group (**Figures 11B–D**). finally, we used western blotting to prove that the expression of the molecules CD155 and PD-L1 was inhibited in the interference group (**Figure 11E**).

## Discussion

In the past, genes that can encode proteins have always been the focus of research on HCC cell mechanisms. However, with the rapid development of experimental technology, noncoding lncRNAs have been proven to be involved in tumor progression and have become a new research topic (19). We identified the disease and molecular regulatory mechanisms, providing a basis for biomedical functional research and the design of more specific molecular biology experiments. In this study, we constructed a new marker using five immune lncRNAs from patients with HCC, and analysis showed that this technique could also independently predict OS. In addition, we used bioinformatics techniques to analyse the molecular mechanisms related to our signatures and the relationship between our model and immune cells. These results suggest that our markers are important in evaluating the prognosis of patients with HCC.

In this research, we obtained five immune lncRNAs related to clinical features from samples: AC009005.1, AC129492.1, AC099850.3, AL365203.2, and AC015908.3. AC129492.1 and AL365203.2 are the first to be reported in HCC, while AC009005.1, AC099850.3, and AC015908.3 have been researched in other cancers. AC009005.1 was relevant to the prognosis of HCC and may be involved in the regulatory mechanism of autophagy of lncRNAs (20). AC099850.3 is not only related to the prognosis and autophagy of HCC but also affects the prognosis of squamous cell carcinoma of the tongue (21). AC015908.3 may be involved in metabolic processes, fibrinolysis and complement activation in HCC to affect prognosis (22).

An important finding of the research was that these immune-related lncRNAs were related to survival in HCC. Our analysis showed that there was an obvious correlation between these molecules and survival, and the high-risk and low-risk groups for OS also showed significant differences, suggesting that these five immune-related lncRNAs were an important prognostic index for HCC. To study the possible mechanism of these five lncRNAs, we carried out further bioinformatics analysis. Combined with the correlation between tumor characteristics and clinicopathological factors, the model could be employed as a predictor of prognosis in patients with HCC. Moreover, the relationship between the signature and survival may indicate that the difference in tumor infiltration may affect the OS of patients. Therefore, this method may be a potential way to predict OS in patients.

The relationship between immune cells and cancer cells in tumors is very complex and is highly related to immune cells involved in tumor promotion and antitumor effects. In this study, we employed CIBERSORT analysis to infer the ratio of 22 immune cells from immune-related genes (23). Comparing HCC tissues with normal tissues, we found that there were significant differences in (16) immune cells, including memory B cells, plasma cells, CD8 T cells, naive CD4 T cells, active memory CD4 T cells, follicular helper T cells, regulatory T cells, resting NK cells, active NK cells, monocytes, M0 macrophages, M1 macrophages, M2 macrophages, resting mast cells and neutrophils. We also found that five kinds of immune cells were significantly positively related to the signature, including activated mast cells, M0 macrophages, resting dendritic cells, and activated memory CD4 T cells. It has been reported that tumor infiltration by T and B cells can improve the survival rate of patients with HCC (24–27). Macrophages in HCC are linked to a good prognosis of HCC, and low immune cell infiltration in hepatocellular carcinoma is associated with the presence of neutrophils, NK cells and resting mast cells (28–31). These results suggest that the 5 lncRNAs in the model may play an important role in the immune cell infiltration of HCC.

Then, the five lncRNAs were used for GO and KEGG enrichment analysis to explore further functions. GO (Gene Ontology) analysis showed that these genes were mostly enriched in “cell cycle checkpoint”, “chromosomal region”, and “DNA replication origin binding”, and the genes were also proven to be actively involved in the cell cycle according to KEGG enrichment analysis. Cell cycle dysfunction is a tumor marker that causes abnormal cell development, and it plays an important role in the occurrence and development of cancer (32). The results suggested that these pathways are related to tumor progression and may account for the mechanism of HCC and offer further ideas for research.

To verify this function, we selected the top coexpressed lncRNA, AC099850.3, for further study. The results showed that AC099850.3 could promote the migration and proliferation of hepatocellular carcinoma cells *in vitro*, and RT-PCR experiments found that AC099850.3 could promote the expression of the cell cycle molecules BUB1, CDK1, PLK1, and TTK.

BUB1 promotes the occurrence and development of HCC by activating mTORC1-related proteins (33). PLK1 and CDK1 are important molecules in the abnormal cell cycle of HCC cells (34). TTK activates the AKT signalling pathway and promotes the progression of HCC cells (35). Then, we used western blotting to prove that the expression of the molecules CD155 and PD-L1 was inhibited in the interference group. CD155 and PDL-1 absence on host and tumor cells exerted an even greater inhibition of tumor growth and metastasis (36, 37). This result suggested that AC099850.3 was related to tumor progression, which might explain the immune mechanism of HCC and provide further ideas for research.

In our research, some limitations should still be recognized. First, although we downloaded samples from multiple databases, the amount of data is still limited. Thus, the clinicopathological parameters analysed in the research may be limited, so we should verify the conclusions in more databases. Finally, there is a lack of basic experiments and clinical trials for further verification of the findings in HCC.

In summary, we established five-immune-related lncRNA-based risk characteristics and proved that they could independently predict the survival of HCC. The model was related to many aspects of the microenvironment of HCC and provides a potential direction for research on immunotherapy for HCC. Therefore, our study may provide a method for HCC therapy in the future.

## Data Availability Statement

The datasets presented in this study can be found in online repositories. The names of the repository/repositories and accession number(s) can be found in the article/**Supplementary Material**.

## Author Contributions

FW and HW analyzed and interpreted the data, and were major contributors in writing the manuscript. GL provided technical support. YZ, who provided ideas and financial support, was our corresponding author. All authors contributed to the article and approved the submitted version.

## Funding

This work was supported by the Science and Technique Innovation Project of Lanzhou University Second Hospital (CY2018-MS14). 2. Special Fund for Doctoral Training of the Lanzhou University Second Hospital (YJS-BD-32).

## Conflict of Interest

The authors declare that the research was conducted in the absence of any commercial or financial relationships that could be construed as a potential conflict of interest.

## Supplementary Material

Supplementary Figure 1 Development of the prognostic signature based on seven immune-related genes. **(A, B)** LASSO regression identified five immune-related lncRNAs in the training dataset.

Supplementary Figure 2 Relationship between the model and clinical characteristics in the training dataset **(A–C)**. Relationship between the model and clinical characteristics in the test dataset **(D–F)**.

Supplementary Table 1 All the primer sequences of the article.

Supplementary Table 2 The result of univariate analysis.

Supplementary Table 3 multivariate Cox regression analysis.

Supplementary Table 4 The lncRNA coexpression results.
