# Supplementary figures and images for: Bioinformatics Profiling of Five Immune-Related lncRNAs for a Prognostic Model of Hepatocellular Carcinoma
Source: Front Oncol. 2021 May 28;11:667904. doi: 10.3389/fonc.2021.667904 (PMC8195283; doi:10.3389/fonc.2021.667904)

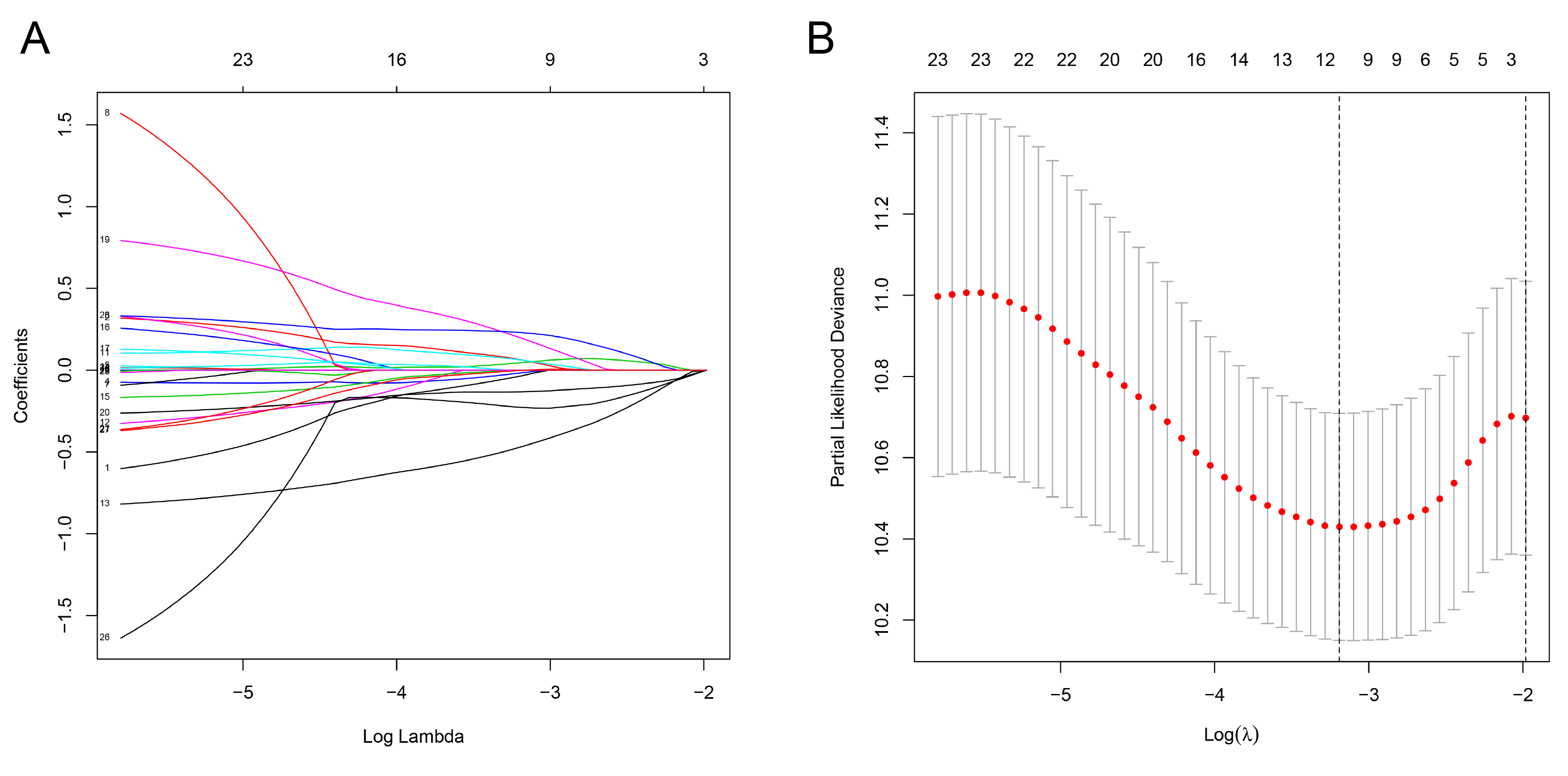

Supplement: Supplementary Figure 1 — Development of the prognostic signature based on seven immune-related genes. (A, B) LASSO regression identified five immune-related lncRNAs in the training dataset. [file Image_1.tif]

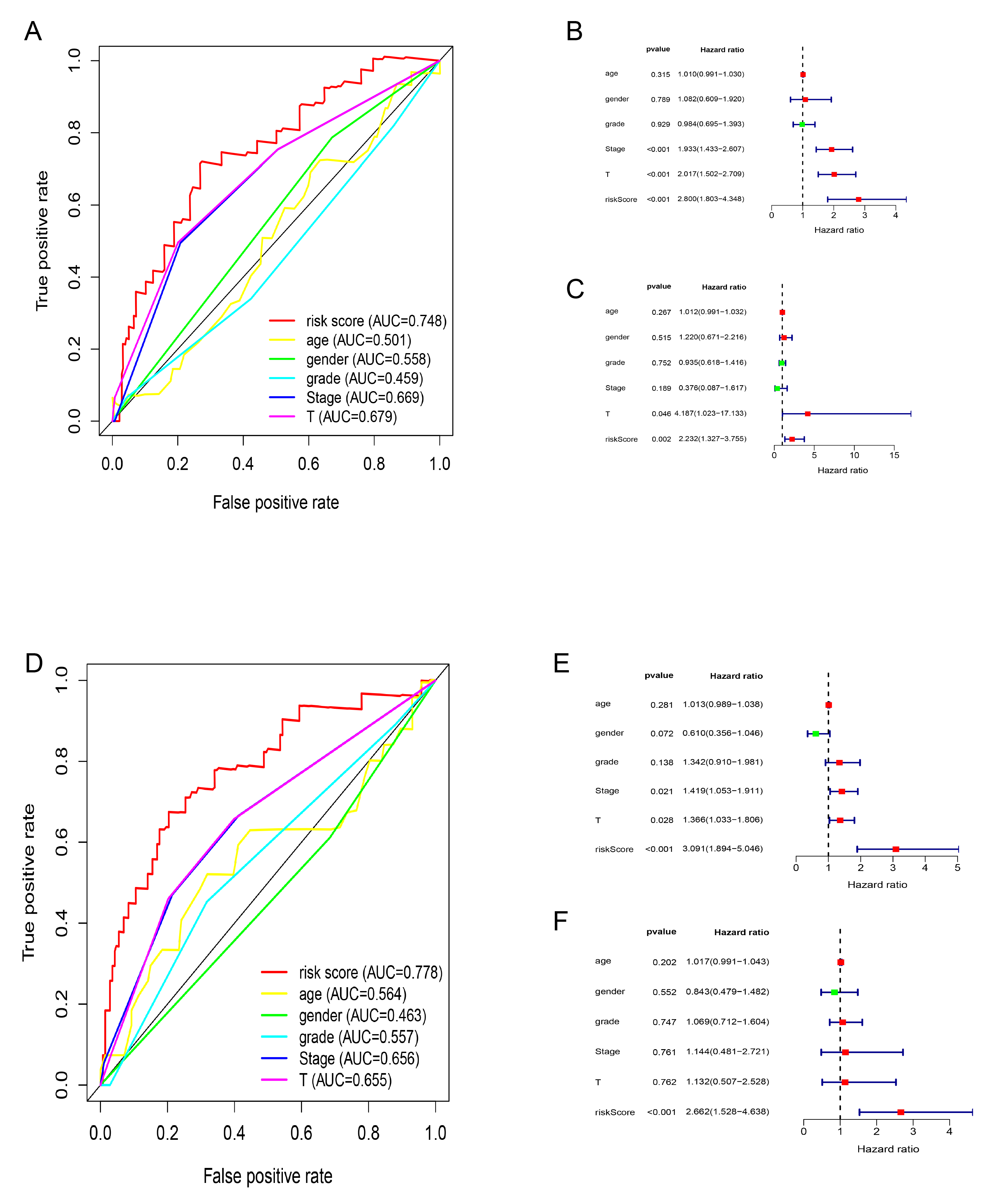

Supplement: Supplementary Figure 2 — Relationship between the model and clinical characteristics in the training dataset (A–C). Relationship between the model and clinical characteristics in the test dataset (D–F). [file Image_2.tif]
