# Supplementary material for: Bioinformatics Profiling of Five Immune-Related lncRNAs for a Prognostic Model of Hepatocellular Carcinoma
Source: Front Oncol. 2021 May 28;11:667904. doi: 10.3389/fonc.2021.667904 (PMC8195283; doi:10.3389/fonc.2021.667904)
Supplement: Supplementary Table 1 — All the primer sequences of the article. [file Table_1.docx]

Table S1. All the primer sequences of the article.

AC099850.3 forward: 5′-TCGCTATGTTTCCCAGGCTGTATT-3′,

reverse: 5′-TGCCAAGGAATCTCTGAAGTCCAT-3′;

BUB1 forward: 5′-ACTGGGAATGGTTCAGGCAA-3′,

reverse:5′-CTGGCTCCTGTGGGTTTATTTT-3′;

CDK1 forward: 5′-CAGTCTTCAGGATGTGCTTATGC-3′,

reverse: 5′-TGTACTGACCAGGAGGGATAGAAT-3′;

MCM2 forward: 5′-CCCAGAACCAGGAGGTGAAA-3′,

reverse: 5′-AGGATGACAGTGGCAAAGACAG-3′; PLK1 forward: 5′-CGACTTCGTGTTCGTGGTGT-3′,

reverse: 5′-GATGAATAACTCGGTTTCGGTG-3′;

TTK forward: 5′-CAGTCATGCCCATTTGGAAGAG-3′,

reverse: 5′-CCACTTGGTTTAGATCCAGGCAC-3′;

β-actin forward: 5′-CACCCAGCACAATGAAGATCAAGAT-3′,

reverse: 5′-CCAGTTTTTAAATCCTGAGTCAAGC-3′.

CD155 forward 5′-ACTGTCACCAGCCTCTGGAT-3′

reverse5′-GGTGAGGTTCACAGTCAGCA-3′.

PDL1 forward: 5′-TTGCTGAACGCCCCATACAA-3

reverse: 5′-GTCCAGATGACTTCGGCCTT-3
