# Supplementary material for: Bioinformatics Profiling of Five Immune-Related lncRNAs for a Prognostic Model of Hepatocellular Carcinoma
Source: Front Oncol. 2021 May 28;11:667904. doi: 10.3389/fonc.2021.667904 (PMC8195283; doi:10.3389/fonc.2021.667904)
Supplement: Supplementary Table 2 — The result of univariate analysis. [file Table_2.docx]

| lncRNA | HR | HR.95L | HR.95H | P value |
| --- | --- | --- | --- | --- |
| AC009005.1 | 1.916095696 | 1.324587779 | 2.771747386 | 0.000555855 |
| CR936218.2 | 0.569481049 | 0.411485907 | 0.788140395 | 0.000683896 |
| AL445493.3 | 0.508644523 | 0.340413575 | 0.760014493 | 0.00096937 |
| TMEM220-AS1 | 0.451801422 | 0.31755444 | 0.642801672 | 1.00E-05 |
| MIR210HG | 1.968820319 | 1.396953543 | 2.774790521 | 0.000109105 |
| AC129492.1 | 0.280246792 | 0.162135101 | 0.484400135 | 5.21E-06 |
| AC099850.3 | 1.847397013 | 1.321853383 | 2.581886742 | 0.000325952 |
| AP001065.1 | 0.527037265 | 0.397635212 | 0.698550505 | 8.36E-06 |
| LINC02362 | 0.619752721 | 0.468635024 | 0.819600362 | 0.000793601 |
| LINC02499 | 0.620320647 | 0.484456121 | 0.794288043 | 0.000153132 |
| AL365203.2 | 2.393412315 | 1.488776423 | 3.8477386 | 0.00031477 |
| AC015908.3 | 0.410461246 | 0.288864024 | 0.583244781 | 6.77E-07 |
| F11-AS1 | 0.531442532 | 0.382943012 | 0.737527925 | 0.000156307 |

Table S2. The result of univariate analysis.
