# Supplementary material for: Bioinformatics Profiling of Five Immune-Related lncRNAs for a Prognostic Model of Hepatocellular Carcinoma
Source: Front Oncol. 2021 May 28;11:667904. doi: 10.3389/fonc.2021.667904 (PMC8195283; doi:10.3389/fonc.2021.667904)
Supplement: Supplementary Table 3 — multivariate Cox regression analysis. [file Table_3.docx]

| lncRNA | coef | HR | HR.95L | HR.95H | P value |
| --- | --- | --- | --- | --- | --- |
| AC009005.1 | 0.517143489 | 1.677229774 | 0.990687115 | 2.839544063 | 0.044212705 |
| AC129492.1 | -0.963749727 | 0.381459831 | 0.170533602 | 0.85327232 | 0.018962639 |
| AC099850.3 | 0.473506769 | 1.605614852 | 0.953962963 | 2.702410003 | 0.044661346 |
| AL365203.2 | 0.738847753 | 2.09352187 | 1.089507143 | 4.022767404 | 0.026608154 |
| AC015908.3 | -1.844052287 | 0.158175154 | 0.043479898 | 0.57542406 | 0.005130596 |

Table S3. The result of multivariate Cox regression analysis
