# Supplementary material for: Bioinformatics Profiling of Five Immune-Related lncRNAs for a Prognostic Model of Hepatocellular Carcinoma
Source: Front Oncol. 2021 May 28;11:667904. doi: 10.3389/fonc.2021.667904 (PMC8195283; doi:10.3389/fonc.2021.667904)
Supplement: Supplementary Table 4 — The lncRNA coexpression results. [file Table_4.docx]

Table S4. The lncRNA coexpression results

| lncRNA | gene | cor | P value |
| --- | --- | --- | --- |
| AC099850.3 | NCAPG | 0.774170034 | 6.97E-76 |
| AC015908.3 | NCAPG | -0.451767494 | 3.29E-20 |
| AC099850.3 | CENPH | 0.540860439 | 8.44E-30 |
| AC099850.3 | RMI2 | 0.47698913 | 1.21E-22 |
| AC099850.3 | RAD54L | 0.75460262 | 4.69E-70 |
| AC015908.3 | RAD54L | -0.505846717 | 1.08E-25 |
| AC099850.3 | NEK2 | 0.757689635 | 6.14E-71 |
| AC099850.3 | PBK | 0.72757839 | 7.44E-63 |
| AC099850.3 | DLGAP5 | 0.78153546 | 3.13E-78 |
| AC015908.3 | DLGAP5 | -0.501656289 | 3.13E-25 |
| AC099850.3 | RACGAP1 | 0.797631335 | 1.07E-83 |
| AC015908.3 | RACGAP1 | -0.488943267 | 7.15E-24 |
| AC129492.1 | F2RL3 | 0.47276081 | 3.19E-22 |
| AC099850.3 | TTK | 0.775991696 | 1.87E-76 |
| AC015908.3 | TTK | -0.452235422 | 2.98E-20 |
| AC099850.3 | FAM83D | 0.576679002 | 1.56E-34 |
| AC099850.3 | RBP7 | -0.478539349 | 8.41E-23 |
| AC015908.3 | RBP7 | 0.457610725 | 9.35E-21 |
| AC099850.3 | TOP2A | 0.840563147 | 4.50E-101 |
| AC015908.3 | TOP2A | -0.514110251 | 1.28E-26 |
| AC099850.3 | SKA1 | 0.657868839 | 9.84E-48 |
| AC099850.3 | NCAPH | 0.817216137 | 4.72E-91 |
| AC015908.3 | NCAPH | -0.54210113 | 5.91E-30 |
| AC099850.3 | DEPDC1 | 0.761502854 | 4.79E-72 |
| AC099850.3 | CENPA | 0.695424261 | 2.43E-55 |
| AC015908.3 | CENPA | -0.454637041 | 1.78E-20 |
| AC099850.3 | CHEK1 | 0.613426459 | 4.93E-40 |
| AC099850.3 | CDC6 | 0.699090918 | 3.80E-56 |
| AC015908.3 | CDC6 | -0.455818155 | 1.38E-20 |
| AC099850.3 | CENPF | 0.807286581 | 3.23E-87 |
| AC015908.3 | CENPF | -0.466358755 | 1.36E-21 |
| AC099850.3 | TPX2 | 0.813131648 | 1.90E-89 |
| AC015908.3 | TPX2 | -0.497574255 | 8.66E-25 |
| AC099850.3 | CHAF1B | 0.608696736 | 2.77E-39 |
| AC099850.3 | NDC80 | 0.690081883 | 3.46E-54 |
| AC099850.3 | FEN1 | 0.617395108 | 1.14E-40 |
| AC099850.3 | RAD51AP1 | 0.780551573 | 6.52E-78 |
| AC099850.3 | DNASE1L3 | -0.49586594 | 1.32E-24 |
| AC015908.3 | DNASE1L3 | 0.471946425 | 3.84E-22 |
| AC099850.3 | PLK1 | 0.771469579 | 4.81E-75 |
| AC015908.3 | PLK1 | -0.518197098 | 4.36E-27 |
| AC099850.3 | TRAIP | 0.49856354 | 6.78E-25 |
| AC099850.3 | GTSE1 | 0.782945279 | 1.09E-78 |
| AC015908.3 | GTSE1 | -0.46730299 | 1.10E-21 |
| AC099850.3 | CCNF | 0.679177654 | 6.55E-52 |
| AC099850.3 | DEPDC1B | 0.645998284 | 1.50E-45 |
| AC015908.3 | DEPDC1B | -0.537015589 | 2.52E-29 |
| AC015908.3 | SLC6A13 | 0.521001598 | 2.06E-27 |
| AC099850.3 | FAM111B | 0.607349884 | 4.49E-39 |
| AC009005.1 | RNASEH2A | 0.465682343 | 1.58E-21 |
| AC099850.3 | RNASEH2A | 0.452519132 | 2.80E-20 |
| AC099850.3 | RRM2 | 0.700882637 | 1.52E-56 |
| AC099850.3 | CDT1 | 0.589270987 | 2.44E-36 |
| AC099850.3 | TK1 | 0.560980991 | 2.18E-32 |
| AC099850.3 | ASPM | 0.72761633 | 7.28E-63 |
| AC099850.3 | PAQR4 | 0.512872377 | 1.77E-26 |
| AC099850.3 | CDC20 | 0.684939304 | 4.22E-53 |
| AC015908.3 | CDC20 | -0.475752133 | 1.60E-22 |
| AC099850.3 | MCM6 | 0.707340585 | 5.25E-58 |
| AC099850.3 | KIF4A | 0.795249091 | 7.42E-83 |
| AC015908.3 | KIF4A | -0.479986663 | 6.00E-23 |
| AC099850.3 | ANLN | 0.847702587 | 1.83E-104 |
| AC015908.3 | ANLN | -0.527614386 | 3.44E-28 |
| AC099850.3 | CCL14 | -0.527315704 | 3.74E-28 |
| AC015908.3 | CCL14 | 0.520495278 | 2.36E-27 |
| AC099850.3 | PRR11 | 0.82151508 | 8.70E-93 |
| AC015908.3 | PRR11 | -0.485972363 | 1.46E-23 |
| AC099850.3 | APOC4 | -0.490737818 | 4.63E-24 |
| AC015908.3 | APOC4 | 0.510423872 | 3.34E-26 |
| AC099850.3 | MKI67 | 0.760552752 | 9.08E-72 |
| AC015908.3 | MKI67 | -0.462655416 | 3.09E-21 |
| AC099850.3 | CDC25C | 0.56499943 | 6.32E-33 |
| AC099850.3 | MAD2L1 | 0.720496835 | 4.14E-61 |
| AC099850.3 | CDC7 | 0.637711323 | 4.39E-44 |
| AC099850.3 | HJURP | 0.73774888 | 1.85E-65 |
| AC015908.3 | HJURP | -0.450353853 | 4.45E-20 |
| AC099850.3 | MYBL2 | 0.682621095 | 1.28E-52 |
| AC015908.3 | MYBL2 | -0.5125285 | 1.94E-26 |
| AC099850.3 | KIF2C | 0.723410953 | 8.05E-62 |
| AC015908.3 | KIF2C | -0.505063776 | 1.32E-25 |
| AC099850.3 | PYURF | -0.516871052 | 6.20E-27 |
| AC099850.3 | CCNA2 | 0.659676087 | 4.49E-48 |
| AC099850.3 | UHRF1 | 0.735874299 | 5.70E-65 |
| AC015908.3 | UHRF1 | -0.473890784 | 2.46E-22 |
| AC099850.3 | TRIP13 | 0.722894865 | 1.08E-61 |
| AC015908.3 | TRIP13 | -0.543415535 | 4.05E-30 |
| AC099850.3 | E2F8 | 0.765656279 | 2.81E-73 |
| AC099850.3 | KIF11 | 0.798929657 | 3.70E-84 |
| AC015908.3 | KIF11 | -0.483198382 | 2.82E-23 |
| AC099850.3 | ZWINT | 0.735558576 | 6.88E-65 |
| AC099850.3 | EXO1 | 0.712786133 | 2.87E-59 |
| AC099850.3 | DTL | 0.680791321 | 3.06E-52 |
| AC099850.3 | UBE2C | 0.646235087 | 1.36E-45 |
| AC099850.3 | CDCA5 | 0.657417318 | 1.20E-47 |
| AC099850.3 | CENPW | 0.50638908 | 9.43E-26 |
| AC099850.3 | GINS1 | 0.767313701 | 8.92E-74 |
| AC015908.3 | GINS1 | -0.488024735 | 8.91E-24 |
| AC099850.3 | SMC2 | 0.683653133 | 7.83E-53 |
| AC099850.3 | MELK | 0.77604394 | 1.80E-76 |
| AC015908.3 | MELK | -0.506816368 | 8.45E-26 |
| AC099850.3 | IQGAP3 | 0.488053698 | 8.85E-24 |
| AC099850.3 | FOXM1 | 0.858416549 | 6.78E-110 |
| AC015908.3 | FOXM1 | -0.485961084 | 1.46E-23 |
| AC099850.3 | CA5A | -0.467986944 | 9.42E-22 |
| AC015908.3 | CA5A | 0.467954813 | 9.49E-22 |
| AC099850.3 | HMMR | 0.742247607 | 1.19E-66 |
| AC009005.1 | TROAP | 0.460459185 | 5.02E-21 |
| AC099850.3 | TROAP | 0.637223407 | 5.34E-44 |
| AC099850.3 | ECT2 | 0.813478888 | 1.39E-89 |
| AC015908.3 | ECT2 | -0.555710749 | 1.08E-31 |
| AC099850.3 | CHML | 0.463686413 | 2.46E-21 |
| AC099850.3 | CDKN3 | 0.634429722 | 1.63E-43 |
| AC099850.3 | CHAF1A | 0.570161253 | 1.25E-33 |
| AC099850.3 | CENPU | 0.557024309 | 7.27E-32 |
| AC009005.1 | PTTG1 | 0.518103221 | 4.47E-27 |
| AC099850.3 | PTTG1 | 0.553643304 | 2.01E-31 |
| AC099850.3 | TCF19 | 0.679284251 | 6.23E-52 |
| AC099850.3 | RAD51 | 0.613970289 | 4.04E-40 |
| AC015908.3 | RAD51 | -0.463674416 | 2.47E-21 |
| AC099850.3 | KIF23 | 0.83666343 | 2.73E-99 |
| AC015908.3 | KIF23 | -0.527060092 | 4.01E-28 |
| AC099850.3 | NUSAP1 | 0.776969665 | 9.15E-77 |
| AC015908.3 | NUSAP1 | -0.459059038 | 6.82E-21 |
| AC099850.3 | CCNB2 | 0.734436449 | 1.34E-64 |
| AC015908.3 | CCNB2 | -0.458959782 | 6.97E-21 |
| AC099850.3 | ARHGAP11A | 0.791897876 | 1.08E-81 |
| AC099850.3 | ATAD2 | 0.453903833 | 2.08E-20 |
| AC099850.3 | CDK1 | 0.750349556 | 7.33E-69 |
| AC099850.3 | CDCA8 | 0.801350355 | 4.98E-85 |
| AC015908.3 | CDCA8 | -0.490799152 | 4.56E-24 |
| AC099850.3 | CKAP2 | 0.659648042 | 4.54E-48 |
| AC099850.3 | C17orf53 | 0.589528954 | 2.24E-36 |
| AC099850.3 | CDCA7 | 0.484641636 | 2.00E-23 |
| AC099850.3 | CENPQ | 0.568239276 | 2.30E-33 |
| AC099850.3 | CKAP2L | 0.837442008 | 1.21E-99 |
| AC099850.3 | KIFC1 | 0.755350694 | 2.87E-70 |
| AC015908.3 | KIFC1 | -0.476046321 | 1.50E-22 |
| AC099850.3 | SKA3 | 0.676036649 | 2.84E-51 |
| AC099850.3 | NUF2 | 0.752253238 | 2.16E-69 |
| AC099850.3 | CEP55 | 0.748553934 | 2.30E-68 |
| AC015908.3 | CEP55 | -0.5613854 | 1.93E-32 |
| AC015908.3 | RDH5 | 0.457907746 | 8.76E-21 |
| AC099850.3 | BUB1 | 0.818182617 | 1.94E-91 |
| AC015908.3 | BUB1 | -0.517768084 | 4.89E-27 |
| AC099850.3 | UBE2T | 0.627106298 | 2.85E-42 |
| AC099850.3 | SPC25 | 0.656620787 | 1.69E-47 |
| AC015908.3 | APOC4-APOC2 | 0.451811179 | 3.26E-20 |
| AC099850.3 | KIF20A | 0.801195237 | 5.67E-85 |
| AC015908.3 | KIF20A | -0.508649533 | 5.28E-26 |
| AC099850.3 | MCM10 | 0.781954545 | 2.29E-78 |
| AC015908.3 | MCM10 | -0.475288355 | 1.79E-22 |
| AC099850.3 | WDR76 | 0.640798264 | 1.26E-44 |
| AC099850.3 | FANCE | 0.490133531 | 5.36E-24 |
| AC099850.3 | FANCI | 0.698572865 | 4.95E-56 |
| AC015908.3 | FANCI | -0.468166414 | 9.05E-22 |
| AC099850.3 | CDC25A | 0.576854588 | 1.48E-34 |
| AC099850.3 | KIF18B | 0.82713568 | 3.99E-95 |
| AC015908.3 | KIF18B | -0.4816942 | 4.02E-23 |
| AC099850.3 | ITIH4 | -0.510953387 | 2.91E-26 |
| AC015908.3 | ITIH4 | 0.45478739 | 1.72E-20 |
| AC099850.3 | MSH2 | 0.736424972 | 4.10E-65 |
| AC015908.3 | MSH2 | -0.463682155 | 2.46E-21 |
| AC099850.3 | MCM2 | 0.744744085 | 2.53E-67 |
| AC015908.3 | MCM2 | -0.469520152 | 6.66E-22 |
| AC099850.3 | STIMATE | -0.533605761 | 6.56E-29 |
| AC015908.3 | STIMATE | 0.499716919 | 5.08E-25 |
| AC099850.3 | CDC45 | 0.636623706 | 6.79E-44 |
| AC099850.3 | CDCA4 | 0.597577257 | 1.42E-37 |
| AC099850.3 | PNPLA7 | -0.462893953 | 2.93E-21 |
| AC009005.1 | BIRC5 | 0.469096398 | 7.33E-22 |
| AC099850.3 | BIRC5 | 0.670117144 | 4.31E-50 |
| AC015908.3 | ACY1 | 0.506009479 | 1.04E-25 |
| AC099850.3 | OIP5 | 0.710353781 | 1.06E-58 |
| AC099850.3 | ORC6 | 0.641168074 | 1.09E-44 |
| AC015908.3 | ORC6 | -0.465388405 | 1.69E-21 |
| AC009005.1 | PDE2A | -0.45259995 | 2.76E-20 |
| AC129492.1 | PDE2A | 0.479050777 | 7.46E-23 |
| AC099850.3 | PDE2A | -0.464002463 | 2.29E-21 |
| AC009005.1 | CENPM | 0.479726429 | 6.37E-23 |
| AC099850.3 | CENPM | 0.530374181 | 1.61E-28 |
| AC129492.1 | MEX3A | -0.513475392 | 1.51E-26 |
| AC099850.3 | ASF1B | 0.674716597 | 5.24E-51 |
| AC015908.3 | GSTZ1 | 0.483381453 | 2.70E-23 |
| AC099850.3 | KIF15 | 0.803961807 | 5.55E-86 |
| AC099850.3 | RHNO1 | 0.631186224 | 5.84E-43 |
| AC099850.3 | PRC1 | 0.81429218 | 6.71E-90 |
| AC015908.3 | PRC1 | -0.489795095 | 5.82E-24 |
| AC099850.3 | SGO1 | 0.750892358 | 5.18E-69 |
| AC099850.3 | SAPCD2 | 0.459284874 | 6.49E-21 |
| AC099850.3 | ORC1 | 0.685193369 | 3.73E-53 |
| AC015908.3 | ORC1 | -0.453405618 | 2.32E-20 |
| AC099850.3 | AURKA | 0.562117949 | 1.54E-32 |
| AC099850.3 | CDCA3 | 0.604717758 | 1.15E-38 |
| AC099850.3 | E2F1 | 0.543110356 | 4.42E-30 |
| AC099850.3 | ZBTB12 | 0.570291041 | 1.20E-33 |
| AC099850.3 | AURKB | 0.578983307 | 7.40E-35 |
| AC099850.3 | SHCBP1 | 0.708149554 | 3.43E-58 |
| AC015908.3 | SHCBP1 | -0.49802926 | 7.74E-25 |
| AC099850.3 | TEDC2 | 0.483006446 | 2.95E-23 |
| AC015908.3 | TTC36 | 0.489768226 | 5.86E-24 |
| AC099850.3 | CCNB1 | 0.725629941 | 2.28E-62 |
| AC015908.3 | CCNB1 | -0.479874025 | 6.16E-23 |
| AC099850.3 | BUB1B | 0.845717905 | 1.67E-103 |
| AC015908.3 | BUB1B | -0.548420398 | 9.43E-31 |
